# Subcutaneous Adipose Tissue Transcriptome Highlights Specific Expression Profiles in Severe Pediatric Obesity: A Pilot Study

**DOI:** 10.3390/cells12081105

**Published:** 2023-04-07

**Authors:** Clarissa Berardo, Valeria Calcaterra, Alessia Mauri, Stephana Carelli, Letizia Messa, Francesca Destro, Federica Rey, Erika Cordaro, Gloria Pelizzo, Gianvincenzo Zuccotti, Cristina Cereda

**Affiliations:** 1 Pediatric Clinical Research Center “Romeo ed Enrica Invernizzi”, Department of Biomedical and Clinical Science, University of Milan, 20157 Milan, Italy; 2 Center of Functional Genomics and Rare Diseases, Department of Pediatrics, Buzzi Children’s Hospital, 20154 Milan, Italy; 3 Pediatric and Adolescent Unit, Department of Internal Medicine, University of Pavia, 27100 Pavia, Italy; 4 Department of Pediatrics, Buzzi Children’s Hospital, 20154 Milan, Italy; 5 Department of Electronics, Information and Bioengineering (DEIB), Politecnico di Milano, 20133 Milan, Italy; 6 Surgery Department, Buzzi Children’s Hospital, 20154 Milan, Italy; 7 Department of Biomedical and Clinical Science, University of Milan, 20157 Milan, Italy

**Keywords:** RNA-Seq, childhood obesity, overweight, lipid metabolism, long non-coding RNAs, periumbilical subcutaneous adipose tissue

## Abstract

The prevalence of pediatric obesity is rising rapidly worldwide, and “omic” approaches are helpful in investigating the molecular pathophysiology of obesity. This work aims to identify transcriptional differences in the subcutaneous adipose tissue (scAT) of children with overweight (OW), obesity (OB), or severe obesity (SV) compared with those of normal weight (NW). Periumbilical scAT biopsies were collected from 20 male children aged 1–12 years. The children were stratified into the following four groups according to their BMI z-scores: SV, OB, OW, and NW. scAT RNA-Seq analyses were performed, and a differential expression analysis was conducted using the DESeq2 R package. A pathways analysis was performed to gain biological insights into gene expression. Our data highlight the significant deregulation in both coding and non-coding transcripts in the SV group when compared with the NW, OW, and OB groups. A KEGG pathway analysis showed that coding transcripts were mainly involved in lipid metabolism. A GSEA analysis revealed the upregulation of lipid degradation and metabolism in SV vs. OB and SV vs. OW. Bioenergetic processes and the catabolism of branched-chain amino acids were upregulated in SV compared with OB, OW, and NW. In conclusion, we report for the first time that a significant transcriptional deregulation occurs in the periumbilical scAT of children with severe obesity compared with those of normal weight or those with overweight or mild obesity.

## 1. Introduction

The adipose tissue (AT) is a heterogeneous endocrine organ consisting of several depots distributed throughout the human body [1]. The AT localized in the connective tissue under the skin is termed subcutaneous AT (scAT), while the fat that surrounds the internal organs is the visceral adipose tissue (VAT) [2]. However, the heterogeneity of AT is not limited to its distribution as it also presents differences at the cellular level which ultimately lead to differences in function [1]. In term of function and lifespan, it is possible to differentiate three types of AT: brown adipose tissue (BAT), beige or brite adipose tissue (BeAT), and white adipose tissue (WAT) [3]. BAT, consisting of multilocular mitochondria-rich cells, is abundant in newborns, where it has the principal function of maintaining body temperature, providing energy in the form of heat [3]. BeAT presents features of both brown and white adipocytes [4]. WAT, in contrast, is composed of large unilocular white adipocytes whose role is to store energy as triglycerides. WAT has two main physiological roles: (i) the metabolic function, i.e., it is responsible for lipogenesis, fatty acid uptake, triglyceride synthesis, and lipolysis; and (ii) the endocrine function via the secretion of hormones and cytokines [5]. However, when an abnormal or excessive WAT accumulation occurs, either by hypertrophy in the scAT and/or by hyperplasia in the VAT [6], obesity arises, leading to the impairment of AT functions, and ultimately resulting in chronic inflammation [7].

Obesity is a growing and alarming disease worldwide, and it affects both adults and children. In 2016, the World Health Organization (WHO) estimated that nearly 41 million children below the age of 5 and over 340 million children and adolescents between 5 and 19 years of age were overweight or affected by obesity, respectively [8]. Pediatric obesity, being a multisystemic condition, has potentially deleterious metabolic consequences, including hyperinsulinemia and insulin resistance, type 2 diabetes, and dyslipidemia [9], and it is closely linked to cardiovascular diseases (CVDs) and all-cause mortality in adults. Even though managing obesity constitutes a primary health purpose, no efficient therapeutic strategy exists to date. For this reason, there is a crucial need to keep investigating the molecular mechanisms involved in obesity onset.

RNA sequencing (RNA-seq) is an approach which enables the study of changes in global gene expression profiles and which can highlight the biological processes associated with different conditions [10]. It is fundamentally important to analyze the gene expression changes responsible for AT dysfunction in order to characterize AT pathophysiology. To date, transcriptomic studies focusing on the obese phenotype have been performed on blood or AT, predominantly in adulthood or in animal models. In particular, RNA-seq studies have been carried out on human adipocytes of scAT or VAT to assess obesity-related modifications [11,12,13]. These transcriptomic analyses have highlighted pathways relevant for adipocyte functions, e.g., Rey et al. have highlighted the importance of long non-coding RNAs (lncRNAs) in the adipogenesis process, underlining the emerging role of non-coding epigenomes in the development of specific comorbidities [14,15,16]. However, the characterization of transcriptional changes in children affected by obesity has not yet been undertaken and is currently limited to some microarray platforms [17]. Compared with arrays, the RNA-seq method enables the detection novel or rare transcripts [18]. Thus, the purpose of this study is to explore the transcriptional profiles of periumbilical scAT using the RNA-seq approach in pediatric patients stratified according to their BMI z-scores.

## 2. Materials and Methods

### 2.1. Anthropometric Measurements and Biochemical Profile

The height, weight, pubertal stage, and waist circumference (WC) of each subject was measured, in accordance with other similar studies [19]. BMI was calculated as body weight (kilograms) divided by height (meters squared), and BMI values were transformed into BMI z-scores using the Centers for Disease Control and Prevention (CDC) reference values [20]. Pubertal stages were defined according to Tanner and classified as follows: prepubertal stage = Tanner Stage 1; middle puberty = Tanner Stages 2–3; late puberty = Tanner Stages 4–5 [21].

All patients underwent a blood draw in a fasting state between 8:30 a.m. and 9:00 a.m., and plasma glucose, insulin, triglycerides (TG), and total and HDL cholesterol were analyzed the same morning using standard methods.

As insulin resistance (IR) surrogates, we considered the following:

- Homeostatic model assessment for insulin resistance (HOMA-IR), calculated as insulin resistance  =  (insulin × glucose)/22.5 [22];
- Triglyceride–glucose index (TyG index), evaluated using the formula (ln[fasting triglycerides (mg/dL) × fasting plasma glucose (mg/dL)/2]) [23].

### 2.2. Adipose Tissue Collection

For the study of the gene expression profile, periumbilical subcutaneous adipose tissues (scATs) were surgically collected from 20 prepubertal male children (1–12 years old) who were enrolled at the Buzzi Children’s Hospital (Milano, Italy). The samples were divided into four groups according to the BMI-z scores of the subjects [24]:

- Normal weight (NW) −2 ≤ BMI-z score < 1 (*n* = 7),
- Overweight (OW) 1 ≤ BMI-z score < 2 (*n* = 3),
- Obesity (OB) 3 ≤ BMI-z score ≤ 2 (*n* = 8),
- Severe obesity (SV) BMI-z score > 3 (*n* = 2),

The institutional ethics committee approved the study (MI area 1–12/2016/2020), and it was conducted in accordance with the Helsinki Declaration of 1975, as revised in 2008.

### 2.3. RNA Isolation and Library Preparation

Total RNA was extracted from the SAT using the Trizol reagent (Invitrogen, Carlsbad, CA, USA), according to the manufacturer’s instructions. RNA quantity and integrity were estimated using the Qubit instrument (Invitrogen, Carlsbad, CA, USA) and the Agilent 4200 TapeStation System (Agilent, Santa Clara, CA, USA). RNA libraries were prepared using the CORALL total RNA-Seq library Prep Kit (Lexogen, Vienna, Austria), and the RiboCop rRNA Depletion Kit (Lexogen, Vienna, Austria) was used to remove rRNA. Library quality was assessed with a High Sensitivity D1000 ScreenTape Assay using the 4200 TapeStation System (Agilent, Santa Clara, CA, USA) and quantified using a Qubit dsDNA HS Assay Kit (Invitrogen, Carlsbad, CA, USA). Libraries were sequenced using 75 bp paired-end reads on the Illumina NextSeq 500 platform (Illumina, San Diego, CA, USA).

### 2.4. Bioinformatic Analysis and Quality Assessment of Raw Data

The quality of the raw data output was examined on a FastQC (last accessed on 31 May 2022). The bioinformatic data analysis pipeline processed with FASTQ was generated using an Illumina NextSeq sequencer via unique molecular identifier (UMI) extraction, trimming, alignment, and quality control steps. Because CORALL libraries contain N12 UMIs at the start of Read 1, UMIs were removed in the first step using the UMI tools software. Adapter sequences, poly(A) sequences at the 3′ end of Read 1, and poly(T) sequences at the 5′ end of Read 2 were then trimmed using the Cutadapt software. Subsequent steps to assess gene and transcript intensities were carried out using the STAR software.

For each sequenced sample, the total number of reads sequenced, the number of reads mapped on the GRCh38, and the percentage of reads on target are reported in Supplementary Table S1. The four samples (OB_12, OB_15, NW_22, and NW_23) with a percentage of mapped reads below 70% were excluded from further bioinformatic analyses. Gene and transcript abundance were computed using the FeatureCounts software, with the “stranded forward” option.

A differential expression analysis was performed using R package DESeq.2; coding and non-coding genes were considered differentially expressed and retained for further analysis when |log2 group2/group1)| ≥ 1 and FDR ≤ 0.1 [25]. The raw data obtained from the RNA-seq analysis are deposited in the Gene Expression Omnibus repository with the accession number GSE228892.

The R software was used to generate heatmaps (the heatmap.2 function from the R ggplots package), PCA plots (the prcomp function from the R ggplots package), and volcano plots. An enrichment analysis of the differentially expressed genes (DEGs) was performed using g:Profiler. A gene ontology and functional enrichment analysis of the DEGs was carried out using the webtools Kyoto Encyclopedia of Genes and Genomes (KEGG, http://www.genome.ad.jp/kegg, accessed on 15 December 2022), Reactome, and WikiPathways. A protein–protein interaction (PPI) analysis was performed using STRING. To evaluate global changes in gene expression, a gene set enrichment analysis (GSEA) was carried out using iDEP (integrated differential expression and pathway analysis, http://bioinformatics.sdstate.edu/idep96/, accessed on 15 December 2022).

## 3. Results

Periumbilical subcutaneous adipose tissues (scATs) were collected from Caucasian male children to identify gene expression patterns associated with obesity. All samples were stratified according to the BMI z-scores of the subjects.

### 3.1. Clinical and Biochemical Features of Enrolled Subjects

The clinical and biochemical parameters of the subjects whose AT was included in the RNA-seq analysis are reported in Table 1.

### 3.2. RNA-Seq Data Analysis

#### 3.2.1. Gene Expression Profiling

**Figure 1:**
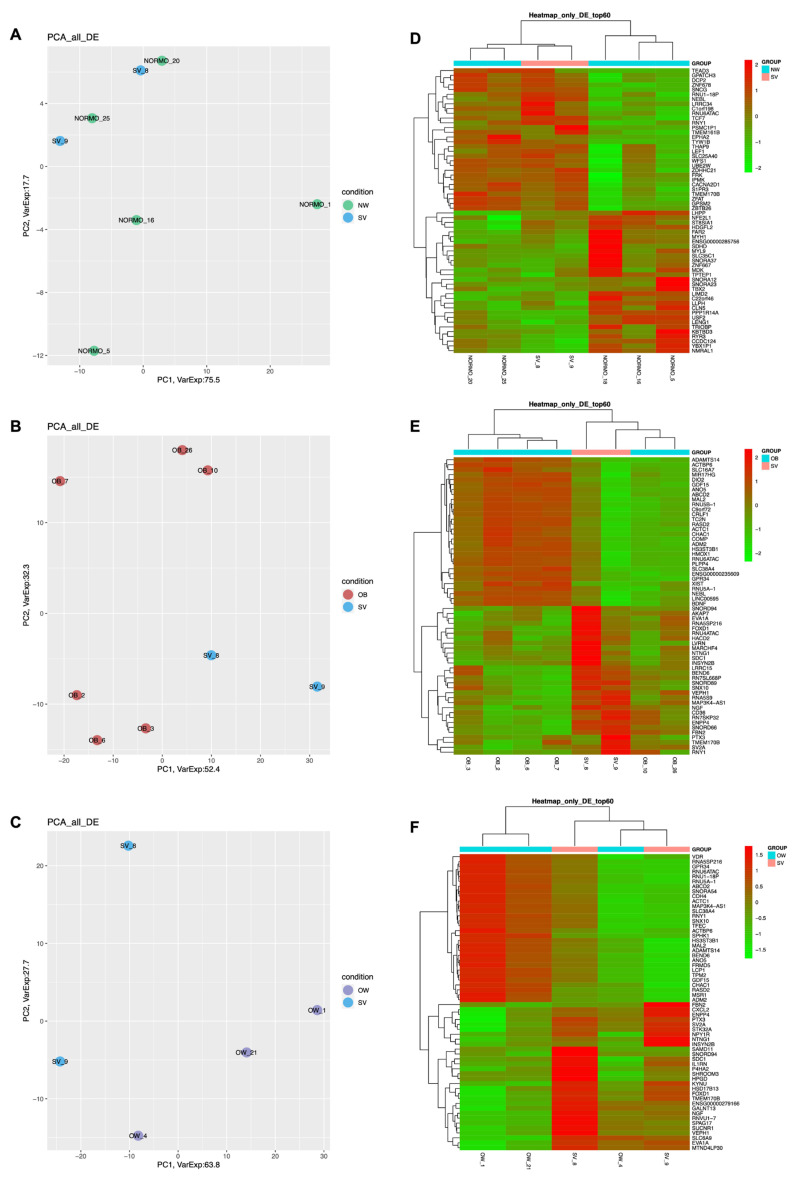
Gene expression profiling. (**A**–**C**): PCA plots obtained from the three comparisons SV vs. NW, SV vs. OB, and SV vs. OW; (**D**–**F**): Heatmaps of SV vs. NW, SV vs. OB, and SV vs. OW. Green: downregulated DEGs; red: upregulated DEGs.

PCA plots summarizing the global variability in gene expression levels allowed us to distinguish specific clusters between OW and NW (Figure S1A), while no difference in cluster distribution was found between OB and OW (Figure S1B) or between SV and NW (Figure 1A). In the SV vs. OB (Figure 1B) and SV vs. OW (Figure 1C) comparisons, specific clusters were observed. The hierarchical clustering heatmaps show the 60 transcripts most deregulated between SV and NW (Figure 1D), SV and OB (Figure 1E), and SV and OW (Figure 1F).

**Figure 2:**
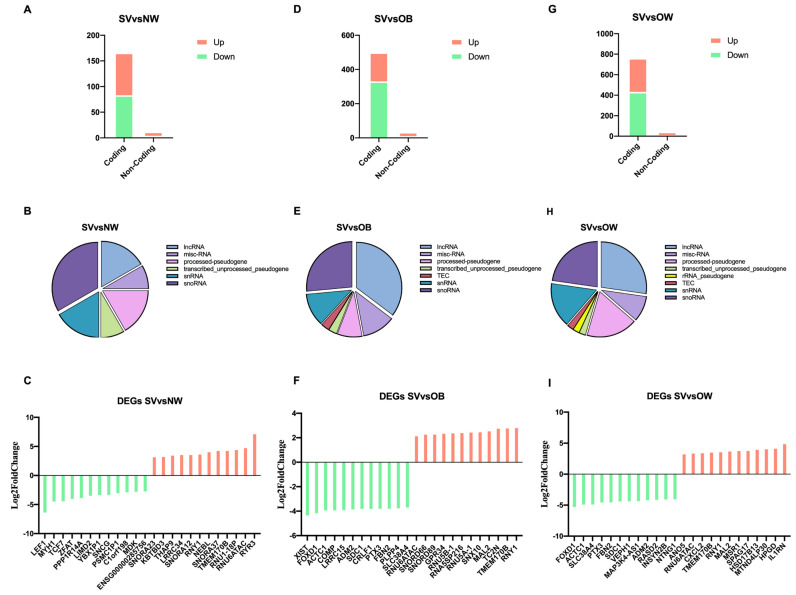
Analysis of RNA biotypes. (**A**,**D**,**G**): bar charts indicating downregulated and upregulated coding and non-coding DEGs found in the SV vs. NW, SV vs. OB, and SV vs. OW comparisons; (**B**,**E**,**H**): pie charts representing the subtypes of non-coding protein DEGs; (**C**,**F**,**I**): bar charts representing the 12 most downregulated and upregulated genes. Green bars represent downregulated DEGs; red bars represent upregulated DEGs.

The comparison between OB and NW showed only 1 downregulated DEG: zinc finger and AT-hook domain containing (ZFAT) (Table S2). In total, three DEGs, including two protein-coding (one downregulated (ZFAT) and one upregulated (odd-skipped related transcription factor 1, OSR1)) and one downregulated lncRNA, were obtained from the comparison between OW and NW (Table S2). There were 178 DEGs between SV and NW, including 166 coding genes (83 downregulated and 83 upregulated) and 12 non-coding genes (4 downregulated and 8 upregulated), as is shown in Figure 2A. Different non-coding biotypes were present, and, specifically, two were long non-coding RNAs (lncRNAs), two were processed pseudogenes, one was a transcribed_unprocessed pseudogene, two were small nuclear RNAs (snRNAs), and four were small nucleolar RNAs (snoRNAs) (Figure 2B). In Figure 2C, the 12 most downregulated and upregulated DEGs in the SV vs. NW comparison are reported. The comparison between OB and OW revealed four downregulated genes, (two coding and two non-coding genes). Of the two non-coding genes, one was a processed pseudogene and one was an unprocessed pseudogene. As is shown in Figure 2C, out of 537 total DEGs between SV and OB, 503 were coding genes (333 downregulated and 170 upregulated) and 34 were non-coding genes (10 downregulated and 24 upregulated). Regarding the non-coding biotypes, 12 were lncRNAs, 3 were processed pseudogenes, 1 was a transcribed_unprocessed pseudogene, 4 were snRNAs, 9 were snRNAs, 1 was a TEC, and 4 were misc_RNAs (Figure 2E). In Figure 2F, the 12 most downregulated and upregulated DEGs in the SV vs. OB comparison are shown. The comparison between SV and OW showed that 762 out of the 796 DEGs were coding genes (432 downregulated and 330 upregulated) and 44 were non-coding genes (11 downregulated and 33 upregulated) (Figure 2G). Regarding the non-coding biotypes reported in Figure 2H, 12 were lncRNAs, 8 were processed pseudogenes, 1 was a transcribed_unprocessed pseudogene, 1 was an rRNA_pseudogene, 7 were snRNAs, 10 were snoRNAs, 1 was a TEC, and 4 were misc_RNAs. In Figure 2I, the 12 most downregulated and upregulated DEGs in the SV vs. OW comparison are reported.

When focusing on lncRNAs, the analysis showed the presence of upregulated and downregulated lncRNAs amongst the different comparisons. Specifically, the SV vs. NW comparison revealed one upregulated lncRNA (OIP5-AS1) and one novel downregulated lncRNA (ENSG00000285756). The SV vs. OB comparison showed six downregulated lncRNAs, of which two had never been reported before (ENSG00000285756 and ENSG00000260267), and five upregulated lncRNAs (ENSG00000261468 and ENSG00000235609 were novel). Finally, in the SV vs. OW comparison, four downregulated lncRNAs were found, of which two were novel (ENSG00000282057 and ENSG00000285756), and seven upregulated lncRNAs were found, of which three were novel (ENSG00000272335, ENSG00000261468, and ENSG00000235609). Notably, OIP5-AS1 and the novel ENSG00000285756 lncRNA were found to be shared in all three comparisons. In contrast, SNHG5, MAP3K4-AS1, and the novel ENSG00000285756, ENSG00000261468, and ENSG00000235609 lncRNAs were found in both the SV vs. OB and SV vs. OW comparisons. Novel lncRNA DEGs are reported in Table 2.

#### 3.2.2. Functional Enrichment Analysis

**Figure 3:**
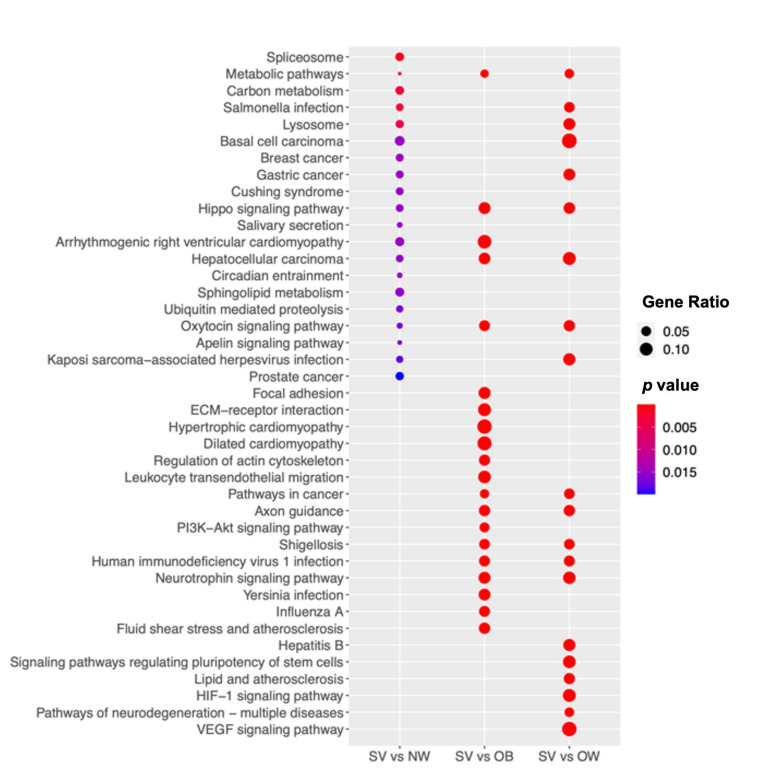
SV group shows significant biologic deregulation. The dot plot shows the KEGG pathway enrichment analyses of the DEGs in SV. The *y*-axis indicates the pathway name and the *x*-axis shows the three comparisons: SV vs. NW, SV vs. OB, and SV vs. OW. The dot size represents the gene ratio and the color bar indicates the adjusted *p*-value (blue represents higher values and red represents lower values).

To gain insight into the biological pathways, a KEGG pathway analysis (Figure 3) together with Reactome and WikiPathways analyses (Figure S2) were performed, comparing the NW, OB, and OW groups with the SV group. The pathways involved in the metabolism of different macromolecules, such as sphingolipids, lipids, and proteins, were found to be deregulated. In addition, the hippo, oxytocin, and apelin signaling pathways were found to be deregulated, as well as the cardiomyopathy pathways (Figure 3). In the PPI analyses, relevant significant networks were only detected among the deregulated genes in the SV vs. OW comparison. In particular, 762 nodes and 1885 edges were predicted, and the GO was significantly enriched, resulting in the primary metabolic process (biological process; GO:0044238; Figure S3).

A KEGG pathway analysis was also conducted for the lncRNAs using the ncPath tool, showing their likely roles in several biological processes. Of particular interest is their involvement in metabolic pathways or pathways linked to events potentially related to excess body weight, e.g., the regulation of lipolysis in adipocytes, the insulin signaling pathway, insulin resistance, type II diabetes mellitus, the GnRH signaling pathway, the estrogen signaling pathway, the thyroid hormone signaling pathway, the TNF signaling pathway, or the IL-17 signaling pathway.

### 3.3. Gene Set Enrichment Analysis

**Figure 4:**
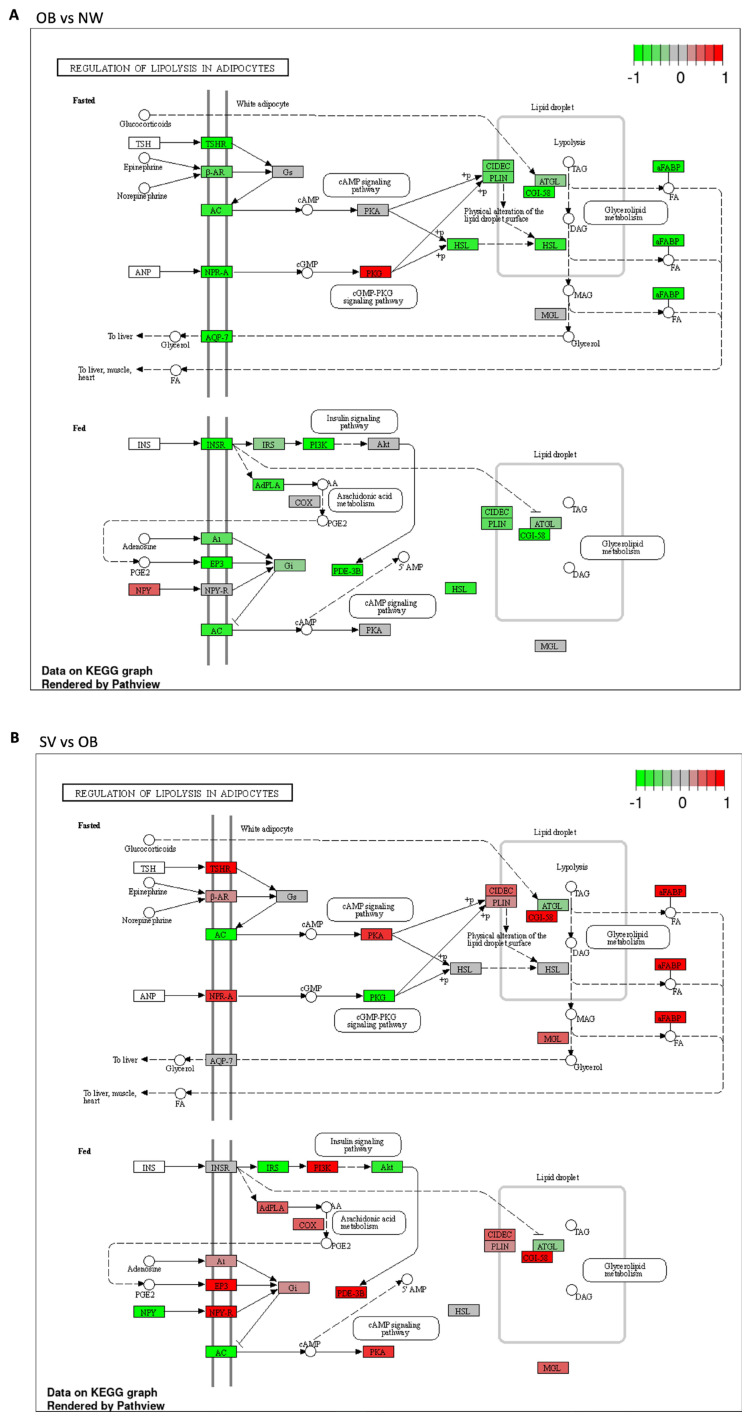
Regulation of lipolysis in adipocytes. (**A**): Schematic representation of the regulation of the lipolysis pathway between OB and NW. Most of the DEGs were downregulated, except for NPY and PKG, which were upregulated; (**B**): Schematic representation of the regulation of lipolysis in SV vs. OB, in which most of the DEGs were upregulated. Green boxes indicate downregulated genes; red boxed indicate upregulated genes; gray boxes indicate genes whose expression is not affected in these conditions.

Further examination of the RNA sequencing data was carried out using GSEA for GO and pathway analysis in order to evaluate global gene expression changes. The downregulated and upregulated pathways shared between the comparisons are reported in Table 3. The lipid metabolism pathways were altered in the SV group when compared with the OB and OW groups. In particular, fatty acid degradation and metabolism were upregulated in SV vs. OB and SV vs. OW, while the regulation of lipolysis in adipocytes was downregulated in OB vs. NW and upregulated in SV vs. OB (Figure 4A,B). Moreover, bioenergetic processes, such as pyruvate metabolism, the TCA cycle, and oxidative phosphorylation, were upregulated in the SV group when compared with the other three groups. In addition, the upregulation of branched-chain amino acid (BCAA) catabolism was observed. Additionally, the NAFLD and myocardiopathy pathways were found to be deregulated in children with severe obesity.

#### 3.3.1. Regulation of Lipolysis in Adipocytes

Regarding the regulation of lipolysis in the adipocyte pathway, the KEGG analysis showed that almost all the proteins involved in this pathway were downregulated (shown in green) in the OB vs. NW comparison (Figure 4A), except for neuropeptide Y (NPY) and protein kinase G (PKG), which were upregulated (shown in red) (Figure 4A). In contrast, in the SV vs. OB comparison, the latter proteins were downregulated (shown in green), together with adenylate cyclase (AC), insulin receptor substrate (IRS), and Akt, while all the others were upregulated (shown in red) (Figure 4B).

#### 3.3.2. Obesity-Associated Diseases

Next, we investigated the gene expression changes in children with severe obesity using the OMIM database in order to assess their possible association with mendelian diseases. Between the SV vs. OB and SV vs. OW comparisons, several OMIM genes associated with dilated cardiomyopathy (VCL, ABCC9, ACTN2, PSEN1, MYH7, DSP, TAZ, FKTN, EYA4, TNNC1, TNNT2, TMPO, LDB3, TNNI3, MYBPC3, TPM1, PSEN2, TTN, ACTC1, LMNA, SGCD, TCAP, DES, SCN5A, PLN, and DMD, all of which were downregulated) and with obesity (SIM1, POMC, LEPR, PPARG, FTO, NTRK2, AKR1C2, PPARGC1B, GHRL, SDC3, ADRB2, LEP, PCSK1, UCP3, UCP2, ADRB3, and ENPP1, all of which were upregulated) were identified.

## 4. Discussion

Childhood obesity represents a serious challenge for global public health [8], and it may also contribute to obesity in adulthood, leading to negative health outcomes. It is known that during obesity, dysfunctional AT undergoes immune, metabolic, and functional changes [26]. To date, microarray analyses of blood cells, isolated adipocytes, adipose tissue, or stromal vascular fractions make up nearly all published studies, and, in some cases, children are grouped together without considering the differences between normal weight and obesity [27]. However, a few RNA-seq studies have been performed on the AT of children or adolescents [28,29]. In their work, Sheldon and colleagues compared intra-abdominal AT collected from severely obese adolescents in relation to different stages of NAFLD [28]. In addition, a pilot study by Zingale et al. focused on the expression of neuro-inflammatory markers, proposing a relationship between obesity and neuro-inflammation [29].

Here, we explored for the first time the transcriptomic profile of scATs collected from the periumbilical area in four different groups of pediatric subjects, showing differences in expression according to the severity of ponderal excess and using BMI as a reliable indicator of body fatness. However, since BMI scores vary with age and sex [20], it was necessary to use BMI z-scores, i.e., BMI normalizations with respect to the two above indicated parameters. Moreover, in our cohort, only prepubertal male children were considered in order to limit the impact of sex hormones, sex chromosome complements, or developmental hormonal variation. It has been demonstrated that during puberty, males and females accumulate different types of lipids [30] in different body regions [31]. In addition, ethnicity can also have an impact on body fat distribution [32], and for this reason our patients were all of European origin.

It is well known that blood levels of triglycerides, cholesterol, glucose, and insulin greatly differ between children with and without obesity [33], rendering these parameters good biomarkers for metabolic derangement. In line with this, blood tests in our patients revealed a pronounced increase in fasting glycemia, insulin, and triglycerides from NW to SV, showing that the differences were already noticeable between NW and OW or OB. However, such changes observed at the plasmatic level were not reflected in the AT gene expression. In fact, no differences were found in the periumbilical scATs of children with either obesity or overweight when compared with those of normal weight, as is shown by the PCA and heatmap plots. Thus, we hypothesize that this could be due to the fact that this type of adipose tissue is less metabolically active than visceral adipose tissue. Thus, it could be interesting to investigate visceral transcriptomic profiles, and to increase the sample size. The most marked differences were observed in the comparisons with the SV group, as demonstrated by the hundreds of coding and non-coding DEGs that were found. The pathway analysis of the coding DEGs revealed their associations with several signaling cascades, including the metabolic, hippo signaling, oxytocin, apelin, and HIF-1α pathways, proving that our data are consistent with previous observations reported in the literature [34,35,36,37]. A recent RNA-seq report on periumbilical scAT from children with obesity and normal weight revealed a significant deregulation in several coding DEGs implied in fatty acid and carbohydrate metabolisms, as well as in inflammatory pathways [29]. In our study, we did not observe any differences between the OB and NW groups. Moreover, even though inflammation is a characteristic feature of obesity [7], no inflammatory pathway was found in any of our comparisons. This may be due to the inflammation likely present in NW subjects undergoing surgery. Moreover, we report a significant variation in non-coding DEGs, highlighting the important impact of epigenetic regulation on obesity. Besides coding genes, a number of non-coding DEGs were also found to be deregulated in the SV group compared with NW, OW, and OB groups. Notably, OIP5-AS1 was found to be upregulated in the SV group when compared with the other groups. Although it has been implicated in a wide variety of cellular processes, as well as in chronic diseases (e.g., diabetes, myocardial ischemia) and the progression of several cancers, we suggest that OIP5-AS1 plays a further role in pediatric obesity [38]. However, further studies are needed to explore the molecular mechanisms involved in its association with obesity, as well as to consolidate the evidence for its potential use in distinguishing children with severe obesity from NW, OW, and OB children.

It is worth emphasizing that even if we notice a significant deregulation between SV and NW, this difference is more pronounced between SV and OB or OW. Obesity is a multifactorial disease, and adipose tissue expansion is a dynamic process that may already have begun during intrauterine life [39]. Adipose tissue alterations depend on several factors, such as eating behaviors, dietary components, the gut microbiome, drugs, and physical exercise [40,41,42]. All of these factors can have epigenetic consequences which impact the adipose tissue gene expression. Moreover, lipids have also been associated with epigenetic regulation, since they can regulate chromatin structure and stability [43,44]. To elucidate the reasons behind the differences in diversity among the groups, it could be very interesting and helpful to study lipidomic profiles.

Furthermore, to achieve a global overview of transcriptional alterations, we compared our data with a priori gene sets. In the SV group, three biological processes were found to be deregulated in comparisons with the other three groups considered in this study: lipid metabolism, bioenergetic processes, and pathways related to CVD. Regarding lipid metabolism, it is interesting to note that while the regulation of lipolysis has been observed to be upregulated in biopsies of the SV group, this pathway was downregulated in the comparison between the OB and NW groups. Insulin was associated with fatty acid synthesis and lipolysis inhibition [45]. However, insulin resistance promotes lipolysis [46]. In line with this, the elevated HOMA-IR index obtained from the SV group seems to correlate with the increase in the lipolysis pathway. However, in the OB group, the lipolysis regulation trend is inverted compared with that of the NW group. It has been reported that TNF-alpha-mediated acute inflammation could accelerate lipolysis [47]. It is important to consider that the NW patients enrolled in this study were subjected to emergency chirurgical interventions, and their results were thus possibly obtained under acute inflammatory conditions. Hence, we suppose that, even though insulin resistance is present in NW children, inflammation could have a major effect on lipolysis regulation. However, more evidence is needed to confirm this supposition. Regarding the bioenergetic processes, it is well known that pyruvate metabolism, the tricarboxylic acid (TCA) cycle, and oxidative phosphorylation are linked together. In fact, the metabolism of pyruvate supports the citric acid cycle thanks to its conversion to acetyl-CoA. The reducing equivalent NADH produced in the TCA is subsequently re-oxidized back into NAD+ in the electron transport chain (ETC) or oxidative phosphorylation, coupling this process with the export of protons across the inner mitochondrial membrane [48]. However, it has been established that these mechanisms are impaired in patients with obesity [49]. For instance, Sohn and colleagues demonstrated that TCA metabolites were higher in children affected by obesity before 18 months of weight loss [50]. The catabolism of branched-chain amino acids (BCCAs) seems to play a central role in all the lipid and energetic pathways. In fact, the degradation of valine, leucine, and isoleucine yields acetyl-CoA molecules, thus feeding the TCA and electron transport chain [51]. However, even though a relationship between BCAAs and obesity has been proved, the functional role of BCAA metabolism in the white adipose tissue of patients suffering from obesity still remains unclear and controversial [52]. In fact, while Green et al. demonstrated that BCAA catabolism increased lipogenesis in adipocyte differentiation [51], here we found an increase in lipolysis and fatty acid degradation. This difference might be due to the fact that our patients were affected by severe obesity, and where therefore in an exacerbated condition with respect to the in vitro model described by Green et al. Lastly, it is known that cardiometabolic risk factors are closely associated with obesity, not only in adulthood, but also in childhood [53]. As expected [54], the triglyceride/HDL cholesterol ratios of our patients lead us to suppose that children with severe obesity are more prone to develop CVD. On the other hand, the RNA-seq data revealed that the cardiomyopathy pathways were deregulated in the SV group, contrary to expectations [53]. However, since scAT has been reported to have fewer metabolic implications than VAT [55], we could hypothesize, in line with the findings of Liu and colleagues [56], that scAT is less associated with cardiovascular risk factors.

The restricted number of enrolled subjects could be considered the main limitation of this study, as it led to the inclusion of only a few samples for each group, in particular for the SV subjects. Even if this condition is very frequent, it is challenging to collect periumbilical subcutaneous adipose tissue from children who do not undergo abdominal surgery, and to harvest biopsies from normal-weight patients is even more challenging. For this reason, here we collected biopsies from patients who needed a surgical intervention not associated with obesity, and the same was done for the normal weight patients. The age of NW subjects could be a second limitation. Hence, we considered children from 1 to 12 years old, avoiding alterations due to breastfeeding (infants >1 year old) or due to puberty (>12 years old). Moreover, we stratified the subjects according to BMI z-score, a parameter that already accounts for the age factor. However, in the future it could be interesting to investigate the expression of the most deregulated genes in NW patients whose ages are comparable with those of the patients in the other groups.

## 5. Conclusions

In conclusion, we report for the first time that a significant transcriptional deregulation occurs in the periumbilical adipose tissue of children with severe obesity when compared with children with overweight or mild obesity, or those of normal weight, with a specific expression profile affecting lipid metabolism. Although this work is to be considered a pilot study and will need to be applied using a wider cohort, it might be useful to integrate our data with other omic approaches.

## Figures and Tables

**Table 1:** Clinical and biochemical features of subjects whose scAT was considered for RNA extraction and RNA-seq study. nv: normal value. *p*-value < 0.001.

Feature	NW *n* = 5	OW *n* = 3	OB *n* = 6	SV *n* = 2
Age	6.87 ± 4.77	10.05 ± 4.23	9.18 ± 2.35	9.90 ± 0.07
Weight (kg)	22.90 ± 11.27	35.76 ± 14.13	47.32 ± 18.22	62.5 ± 0.70
Height (cm)	118.20 ± 3.97	130.0 ± 20.88	134.06 ± 16.31	141.0 ± 1.41
Body mass index (BMI)				
- kg/m^2^ - z-score	15.4 ± 2.27 0.11 ± 1.49	20.3 ± 2.5 1.68 ± 0.17	21.55 ± 2.19 2.63 ± 0.54	31.2 ± 0.56 3.4 ± 0.00
Fasting blood glycemia (mg/dL; nv < 100 mg/dL)	79.0 ± 8.54	84.0 ± 1.81	98.33 ± 4.72	101.0 ± 1.41
Insulin	7.50 ± 0.70	15.5 ± 0.70	16.93 ± 7.04	21.1 ± 0.007
Triglycerides (mg/dL) (nv ≥ 130 mg/dL if ≥ 10 years)	57.33 ± 9.29	67.5 ± 3.53	118.33 ± 21.07	130.5 ± 0.70
HDL cholesterol (mg/dL) (nv > 50 in males)	57.5 ± 3.53	47.0 ± 18.38	53.33 ± 6.65	49.5 ± 0.70
Triglycerides/HDL cholesterol ratio (nv < 2.2)	1.09 ± 0.12	1.57 ± 0.68	2.27 ± 0.63	2.63 ± 0.02
Triglyceride–glucose index (nv < 7.88)	7.75 ± 0.009	7.96 ± 0.03	8.65 ± 0.23	8.79 ± 0.008
HOMA-IR	1.38 ± 0.22	3.25 ± 0.09	4.26 ± 1.85	5.23 ± 0.07

**Table 2:** Novel lncRNAs found in the study. DEGs and their respective expressions (Log2FoldChange) obtained from RNA-seq in the following comparisons: OW vs. NW, SV vs. NW, SV vs. OB, and SV vs. OW.

Comparison	ENSEMBL	Log2FoldChange
OW vs. NW	ENSG00000288900	−5.149906832
SV vs. NW	ENSG00000285756	−2.980591461
SV vs. OB	ENSG00000285756 ENSG00000260267 ENSG00000261468 ENSG00000235609	−1.955798388 −1.111281536 1.348606443 1.747909824
SV vs. OW	ENSG00000282057 ENSG00000285756 ENSG00000272335 ENSG00000261468 ENSG00000235609	−3.180240676 −2.327811635 1.388553119 1.990157997 2.196794819

**Table 3:** GSEA analysis. Pathways deregulated in the SV vs. NW, OB, or OW comparisons. The pathways are listed according to the grouping target involved in the same metabolic path. Green font indicated downregulated pathways; red font indicates upregulated pathways.

	SV vs. NW	SV vs. OB	SV vs. OW
Regulation of lipolysis in adipocytes		**up**	
Fatty acid degradation		**up**	**up**
Fatty acid metabolism		**up**	**up**
PPAR signaling pathway		**up**	**up**
Pyruvate metabolism		**up**	**up**
Citrate cycle (TCA cycle)		**up**	**up**
Oxidative phosphorylation	**up**	**up**	**up**
Valine, leucine, and isoleucine degradation		**up**	**up**
Non-alcoholic fatty liver disease		**up**	**up**
Dilated cardiomyopathy		**down**	**down**
Hypertrophic cardiomyopathy		**down**	**down**
Arrhythmogenic right ventricular cardiomyopathy		**down**	**down**

## Data Availability

The raw data obtained from the RNA-Seq analysis have been deposited in the Gene Expression Omnibus repository (GSE228892).

## Supplementary Materials

The following supporting information can be downloaded at: https://www.mdpi.com/article/10.3390/cells12081105/s1, Figure S1: PCA plots of OW vs. NW and OB vs. OW; Figure S2: Reactome and Wikipathway enrichment pathway analysis; Figure S3: PPI analysis network in SV vs. OW comparison; Table S1: Summary statistics of reads mapping and transcripts assembly for each sample; Table S2: DEGs obtained in the following comparisons: OB vs. NW, OW vs. NW, and OB vs. OW.

## Abbreviations

AT	Adipose tissue
BAT	Brown adipose tissue
BCAA	Branched-chain amino acid
BeAT	Beige adipose tissue
BMI	Body mass index
CDC	Centers for Disease Control and Prevention
CVD	Cardiovascular disease
DEG	Differentially expressed gene
ETC	Electron transport chain
GSEA	Gene set enrichment analysis
HDL	High-density lipoprotein
HOMA-IR	Homeostatic model assessment for insulin resistance
KEGG	Kyoto encyclopedia of genes and genomes
lncRNA	Long non-coding RNA
NAFLD	Non-alcoholic fatty liver disease
NW	Normal weight
OB	Obesity
OW	Overweight
PPI	Protein–protein interaction
RNA-seq	RNA sequencing
scAT	Subcutaneous adipose tissue
snRNA	Small nuclear RNA
snoRNA	Small nucleolar RNA
SV	Severe obesity
VAT	Visceral adipose tissue
TCA	Tricarboxylic acid
WAT	White adipose tissue
WC	Waist circumference
WHO	World Health Organization

## References

[1] Lee M.-J., Wu Y., Fried S.K. (2013). Adipose Tissue Heterogeneity: Implication of Depot Differences in Adipose Tissue for Obesity Complications. Mol. Aspects Med..

[2] Mittal B. (2019). Subcutaneous Adipose Tissue & Visceral Adipose Tissue. Indian J. Med. Res..

[3] Menendez A., Wanczyk H., Walker J., Zhou B., Santos M., Finck C. (2022). Obesity and Adipose Tissue Dysfunction: From Pediatrics to Adults. Genes.

[4] Pilkington A.-C., Paz H.A., Wankhade U.D. (2021). Beige Adipose Tissue Identification and Marker Specificity—Overview. Front. Endocrinol..

[5] Gesta S., Kahn C.R., Symonds M.E. (2017). White Adipose Tissue. Adipose Tissue Biology.

[6] Leyvraz C., Verdumo C., Giusti V. (2008). Localization of adipose tissue: Clinical implications. Rev. Med. Suisse.

[7] Zatterale F., Longo M., Naderi J., Raciti G.A., Desiderio A., Miele C., Beguinot F. (2019). Chronic Adipose Tissue Inflammation Linking Obesity to Insulin Resistance and Type 2 Diabetes. Front. Physiol..

[8] Obesity and Overweight. https://www.who.int/news-room/fact-sheets/detail/obesity-and-overweight.

[9] Chen Y.Y., Wang J.P., Jiang Y.Y., Li H., Hu Y.H., Lee K.O., Li G.W. (2015). Fasting Plasma Insulin at 5 Years of Age Predicted Subsequent Weight Increase in Early Childhood over a 5-Year Period-The Da Qing Children Cohort Study. PLoS ONE.

[10] D’Agostino N., Li W., Wang D. (2022). High-Throughput Transcriptomics. Sci. Rep..

[11] Armenise C., Lefebvre G., Carayol J., Bonnel S., Bolton J., Di Cara A., Gheldof N., Descombes P., Langin D., Saris W.H. (2017). Transcriptome Profiling from Adipose Tissue during a Low-Calorie Diet Reveals Predictors of Weight and Glycemic Outcomes in Obese, Nondiabetic Subjects. Am. J. Clin. Nutr..

[12] Ferguson J.F., Xue C., Hu Y., Li M., Reilly M.P. (2016). Adipose Tissue RNASeq Reveals Novel Gene-Nutrient Interactions Following n-3 PUFA Supplementation and Evoked Inflammation in Humans. J. Nutr. Biochem..

[13] Krekmanov L., Nordenram A. (1986). Postoperative Complications after Surgical Removal of Mandibular Third Molars. Effects of Penicillin V and Chlorhexidine. Int. J. Oral Maxillofac. Surg..

[14] Rey F., Zuccotti G.V., Carelli S. (2021). Long Non-Coding RNAs in Metabolic Diseases: From Bench to Bedside. Trends Endocrinol. Metab. TEM.

[15] Rey F., Messa L., Pandini C., Launi R., Barzaghini B., Micheletto G., Raimondi M.T., Bertoli S., Cereda C., Zuccotti G.V. (2021). Transcriptome Analysis of Subcutaneous Adipose Tissue from Severely Obese Patients Highlights Deregulation Profiles in Coding and Non-Coding Oncogenes. Int. J. Mol. Sci..

[16] Rey F., Messa L., Pandini C., Barzaghini B., Micheletto G., Raimondi M.T., Bertoli S., Cereda C., Zuccotti G.V., Cancello R. (2021). Transcriptional Characterization of Subcutaneous Adipose Tissue in Obesity Affected Women Highlights Metabolic Dysfunction and Implications for LncRNAs. Genomics.

[17] Liu Y., Ji Y., Li M., Wang M., Yi X., Yin C., Wang S., Zhang M., Zhao Z., Xiao Y. (2018). Integrated Analysis of Long Noncoding RNA and MRNA Expression Profile in Children with Obesity by Microarray Analysis. Sci. Rep..

[18] Wang C., Gong B., Bushel P.R., Thierry-Mieg J., Thierry-Mieg D., Xu J., Fang H., Hong H., Shen J., Su Z. (2014). The Concordance between RNA-Seq and Microarray Data Depends on Chemical Treatment and Transcript Abundance. Nat. Biotechnol..

[19] Calcaterra V., Montalbano C., de Silvestri A., Pelizzo G., Regalbuto C., Paganelli V., Albertini R., Cave F.D., Larizza D., Cena H. (2019). Triglyceride Glucose Index as a Surrogate Measure of Insulin Sensitivity in a Caucasian Pediatric Population. J. Clin. Res. Pediatr. Endocrinol..

[20] CDC BMI for Children and Teens. https://www.cdc.gov/obesity/basics/childhood-defining.html.

[21] Marshall W.A., Tanner J.M. (1969). Variations in Pattern of Pubertal Changes in Girls. Arch. Dis. Child..

[22] Matthews D.R., Hosker J.P., Rudenski A.S., Naylor B.A., Treacher D.F., Turner R.C. (1985). Homeostasis Model Assessment: Insulin Resistance and Beta-Cell Function from Fasting Plasma Glucose and Insulin Concentrations in Man. Diabetologia.

[23] Simental-Mendía L.E., Rodríguez-Morán M., Guerrero-Romero F. (2008). The Product of Fasting Glucose and Triglycerides as Surrogate for Identifying Insulin Resistance in Apparently Healthy Subjects. Metab. Syndr. Relat. Disord..

[24] de Onis M., Onyango A.W., Borghi E., Siyam A., Nishida C., Siekmann J. (2007). Development of a WHO Growth Reference for School-Aged Children and Adolescents. Bull. World Health Organ..

[25] Messa L., Rey F., Pandini C., Barzaghini B., Micheletto G., Raimondi M.T., Bertoli S., Cereda C., Zuccotti G., Cancello R. (2021). RNA-Seq Dataset of Subcutaneous Adipose Tissue: Transcriptional Differences between Obesity and Healthy Women. Data Brief.

[26] Alexaki V.I. (2021). The Impact of Obesity on Microglial Function: Immune, Metabolic and Endocrine Perspectives. Cells.

[27] Costa A., Reynés B., Konieczna J., Martín M., Fiol M., Palou A., Romaguera D., Oliver P. (2021). Use of Human PBMC to Analyse the Impact of Obesity on Lipid Metabolism and Metabolic Status: A Proof-of-Concept Pilot Study. Sci. Rep..

[28] Sheldon R.D., Kanosky K.M., Wells K.D., Miles L., Perfield J.W., Xanthakos S., Inge T.H., Rector R.S. (2016). Transcriptomic Differences in Intra-Abdominal Adipose Tissue in Extremely Obese Adolescents with Different Stages of NAFLD. Physiol. Genomics.

[29] Zingale V.D., D’Angiolini S., Chiricosta L., Calcaterra V., Selvaggio G.G.O., Zuccotti G., Destro F., Pelizzo G., Mazzon E. (2022). Does Childhood Obesity Trigger Neuroinflammation?. Biomedicines.

[30] Loomba-Albrecht L.A., Styne D.M. (2009). Effect of Puberty on Body Composition. Curr. Opin. Endocrinol. Diabetes Obes..

[31] Adami F., Benedet J., Takahashi L.A.R., da Silva Lopes A., da Silva Paiva L., de Vasconcelos F. (2020). de A.G. Association between Pubertal Development Stages and Body Adiposity in Children and Adolescents. Health Qual. Life Outcomes.

[32] Martos-Moreno G.Á., Martínez-Villanueva J., González-Leal R., Barrios V., Sirvent S., Hawkins F., Chowen J.A., Argente J. (2020). Ethnicity Strongly Influences Body Fat Distribution Determining Serum Adipokine Profile and Metabolic Derangement in Childhood Obesity. Front. Pediatr..

[33] García A.G., Urbina Treviño M.V., Villalpando Sánchez D.C., Aguilar C.A. (2019). Diagnostic Accuracy of Triglyceride/Glucose and Triglyceride/HDL Index as Predictors for Insulin Resistance in Children with and without Obesity. Diabetes Metab. Syndr..

[34] Hong S.M., Ko J.-K., Moon J.-J., Kim Y.-R. (2021). Oxytocin: A Potential Therapeutic for Obesity. J. Obes. Metab. Syndr..

[35] Shen H., Huang X., Zhao Y., Wu D., Xue K., Yao J., Wang Y., Tang N., Qiu Y. (2022). The Hippo Pathway Links Adipocyte Plasticity to Adipose Tissue Fibrosis. Nat. Commun..

[36] Gaspar J.M., Velloso L.A. (2018). Hypoxia Inducible Factor as a Central Regulator of Metabolism—Implications for the Development of Obesity. Front. Neurosci..

[37] Sentinelli F., Bertoccini L., Incani M., Pani M.G., David F., Bailett D., Boi A., Barchetta I., Cimini F.A., Mannino A.C. (2020). Association of Apelin Levels in Overweight-Obese Children with Pubertal Development, but Not with Insulin Sensitivity: 6.5 Years Follow up Evaluation. Endocr. Res..

[38] Wooten S., Smith K.N. (2022). Long Non-Coding RNA OIP5-AS1 (Cyrano): A Context-Specific Regulator of Normal and Disease Processes. Clin. Transl. Med..

[39] Orsso C.E., Colin-Ramirez E., Field C.J., Madsen K.L., Prado C.M., Haqq A.M. (2020). Adipose Tissue Development and Expansion from the Womb to Adolescence: An Overview. Nutrients.

[40] Petridou A., Siopi A., Mougios V. (2019). Exercise in the Management of Obesity. Metabolism.

[41] Gonçalves P., Araújo J.R., Martel F. (2015). Antipsychotics-Induced Metabolic Alterations: Focus on Adipose Tissue and Molecular Mechanisms. Eur. Neuropsychopharmacol..

[42] Derrien M., Alvarez A.-S., de Vos W.M. (2019). The Gut Microbiota in the First Decade of Life. Trends Microbiol..

[43] Zaina S., Døssing K.B., Lindholm M.W., Lund G. (2005). Chromatin Modification by Lipids and Lipoprotein Components: An Initiating Event in Atherogenesis?. Curr. Opin. Lipidol..

[44] Dekkers K.F., Slagboom P.E., Jukema J.W., Heijmans B.T. (2016). The Multifaceted Interplay between Lipids and Epigenetics. Curr. Opin. Lipidol..

[45] Li Y., Li Z., Ngandiri D.A., Llerins Perez M., Wolf A., Wang Y. (2022). The Molecular Brakes of Adipose Tissue Lipolysis. Front. Physiol..

[46] Zhao J., Wu Y., Rong X., Zheng C., Guo J. (2020). Anti-Lipolysis Induced by Insulin in Diverse Pathophysiologic Conditions of Adipose Tissue. Diabetes Metab. Syndr. Obes. Targets Ther..

[47] Rittig N., Bach E., Thomsen H.H., Pedersen S.B., Nielsen T.S., Jørgensen J.O., Jessen N., Møller N. (2016). Regulation of Lipolysis and Adipose Tissue Signaling during Acute Endotoxin-Induced Inflammation: A Human Randomized Crossover Trial. PLoS ONE.

[48] Lagarde D., Jeanson Y., Portais J.-C., Galinier A., Ader I., Casteilla L., Carrière A. (2021). Lactate Fluxes and Plasticity of Adipose Tissues: A Redox Perspective. Front. Physiol..

[49] Masschelin P.M., Cox A.R., Chernis N., Hartig S.M. (2020). The Impact of Oxidative Stress on Adipose Tissue Energy Balance. Front. Physiol..

[50] Sohn M.-J., Chae W., Ko J.-S., Cho J.-Y., Kim J.-E., Choi J.-Y., Jang H.-B., Lee H.-J., Park S.-I., Park K.-H. (2021). Metabolomic Signatures for the Effects of Weight Loss Interventions on Severe Obesity in Children and Adolescents. Metabolites.

[51] Green C.R., Wallace M., Divakaruni A.S., Phillips S.A., Murphy A.N., Ciaraldi T.P., Metallo C.M. (2016). Branched-Chain Amino Acid Catabolism Fuels Adipocyte Differentiation and Lipogenesis. Nat. Chem. Biol..

[52] Ma Q.-X., Zhu W.-Y., Lu X.-C., Jiang D., Xu F., Li J.-T., Zhang L., Wu Y.-L., Chen Z.-J., Yin M. (2022). BCAA-BCKA Axis Regulates WAT Browning through Acetylation of PRDM16. Nat. Metab..

[53] Bendor C.D., Bardugo A., Pinhas-Hamiel O., Afek A., Twig G. (2020). Cardiovascular Morbidity, Diabetes and Cancer Risk among Children and Adolescents with Severe Obesity. Cardiovasc. Diabetol..

[54] Katsa M.E., Ioannidis A., Sachlas A., Dimopoulos I., Chatzipanagiotou S., Rojas Gil A.P. (2019). The Roles of Triglyceride/High-Density Lipoprotein Cholesterol Ratio and Uric Acid as Predisposing Factors for Metabolic Syndrome in Healthy Children. Ann. Pediatr. Endocrinol. Metab..

[55] Ibrahim M.M. (2010). Subcutaneous and Visceral Adipose Tissue: Structural and Functional Differences. Obes. Rev..

[56] Liu J., Fox C.S., Hickson D.A., May W.D., Hairston K.G., Carr J.J., Taylor H.A. (2010). Impact of Abdominal Visceral and Subcutaneous Adipose Tissue on Cardiometabolic Risk Factors: The Jackson Heart Study. J. Clin. Endocrinol. Metab..

