# Supplementary material for: Subcutaneous Adipose Tissue Transcriptome Highlights Specific Expression Profiles in Severe Pediatric Obesity: A Pilot Study
Source: Cells. 2023 Apr 7;12(8):1105. doi: 10.3390/cells12081105 (PMC10137076; doi:10.3390/cells12081105)
Supplement: Supplementary file 1 [file cells-12-01105-s001.zip › Table S1_Summary mapping statistics.pdf]

Table S1: Summary statistics of reads mapping and transcripts assembly for each sample. The gray lines correspond to the sample excluded from further analyses.

Sample	Number of input reads	Uniquely mapped reads number	Uniquely mapped reads %
SV_8	18932671	13682788	72.27%
SV_9	28959345	21581279	74.52%
OB_2	16771250	12616970	75.23%
OB_3	14348495	10997336	76.64%
OB_6	19272092	14020704	72.75%
OB_7	15458160	11225717	72.62%
OB_10	18062296	12876346	71.29%
OB_12	22963574	139842	0.61%
OB_15	12045660	3431262	28.49%
OB_26	20757470	14650064	70.58%
OW_1	22054804	17095535	77.51%
OW_4	19175098	13745116	71.68%
OW_21	31502049	24334717	77.25%
NW_5	28395344	21422848	75.44%
NW_16	19917439	15292906	76.78%
NW_18	23736169	17803283	75.00%
NW_20	19979054	14781939	73.99%
NW_22	28885675	18505813	64.07%
NW_23	12609668	4884161	38.73%
NW_25	30702039	23283207	75.84%
