# Supplementary material for: Subcutaneous Adipose Tissue Transcriptome Highlights Specific Expression Profiles in Severe Pediatric Obesity: A Pilot Study
Source: Cells. 2023 Apr 7;12(8):1105. doi: 10.3390/cells12081105 (PMC10137076; doi:10.3390/cells12081105)
Supplement: Supplementary file 1 [file cells-12-01105-s001.zip › Table S2_DEGs in OBvsNW, OWvsNW, OBvsOW.pdf]

Table S2. DEGs found in the following comparisons: OB vs NW, OW vs NW, and OB vs OW.

Comparison	Gene name	Coding	Log2FoldChange
**OB vs NW**	ZFAT	+	-3,103737829
**OW vs NW**	ENSG00000288900	-	-5,149906832
	ZFAT	+	-2,612986442
	OSR1	+	1,718691559
**OB vs OW**	MTCO2P12	-	3,380818979
	TPSAB1	+	3,619902117
	CMA1	+	4,821164022
	RPL31P57	-	21,43006153
