# Supplementary figures and images for: Subcutaneous Adipose Tissue Transcriptome Highlights Specific Expression Profiles in Severe Pediatric Obesity: A Pilot Study
Source: Cells. 2023 Apr 7;12(8):1105. doi: 10.3390/cells12081105 (PMC10137076; doi:10.3390/cells12081105)

**A**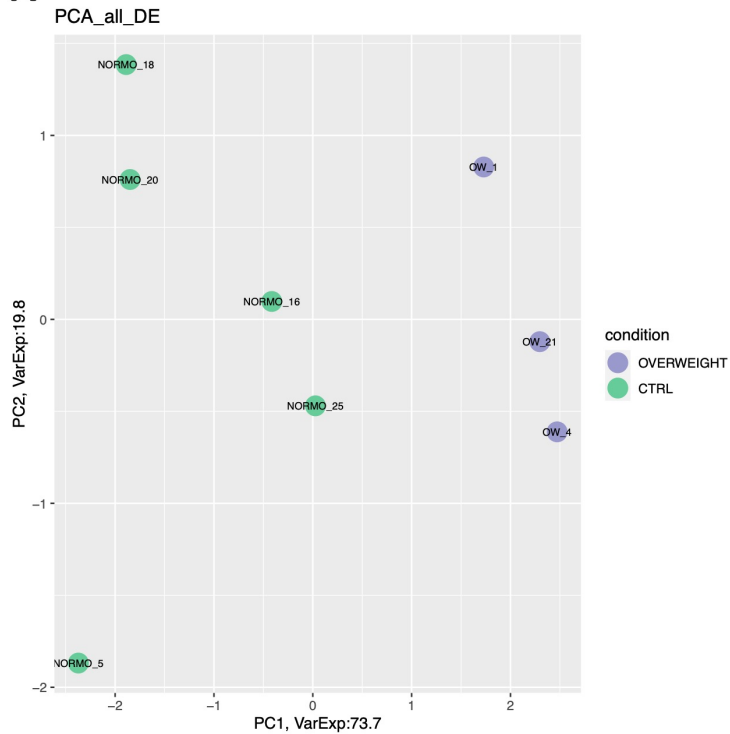**B**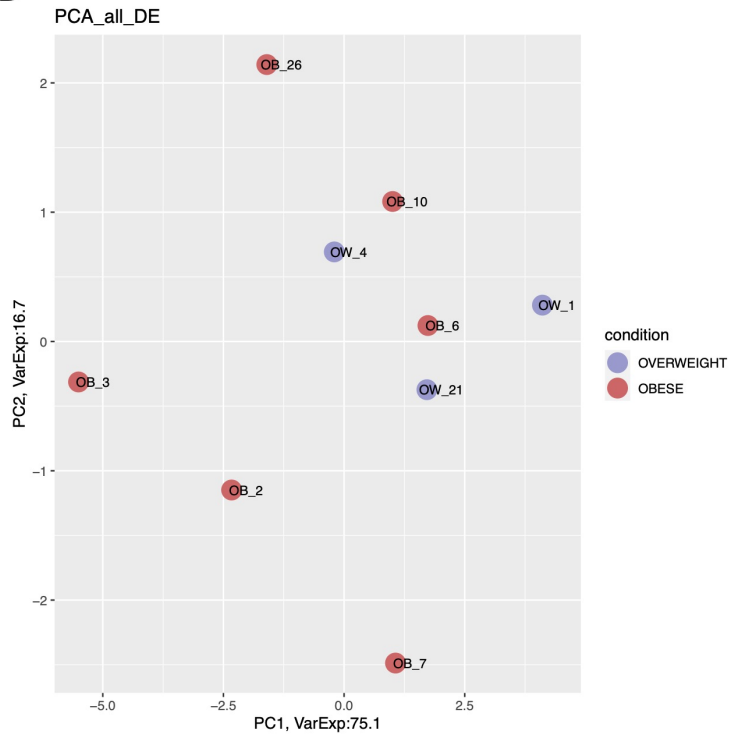

Supplement: Supplementary file 1 [file cells-12-01105-s001.zip › FigureS1_PCA plots OWvsNW, OBvsOW.pdf]

A

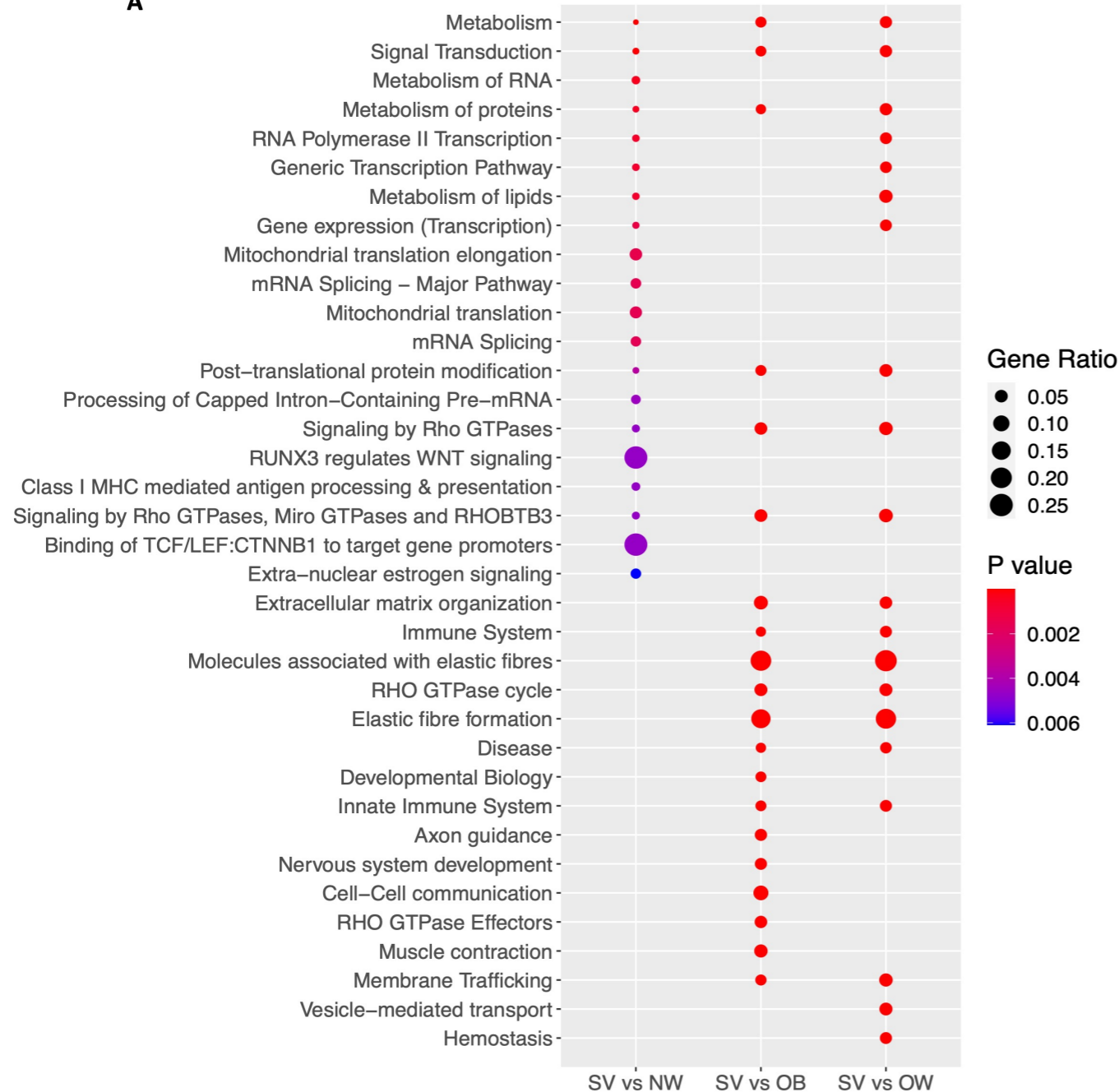

B

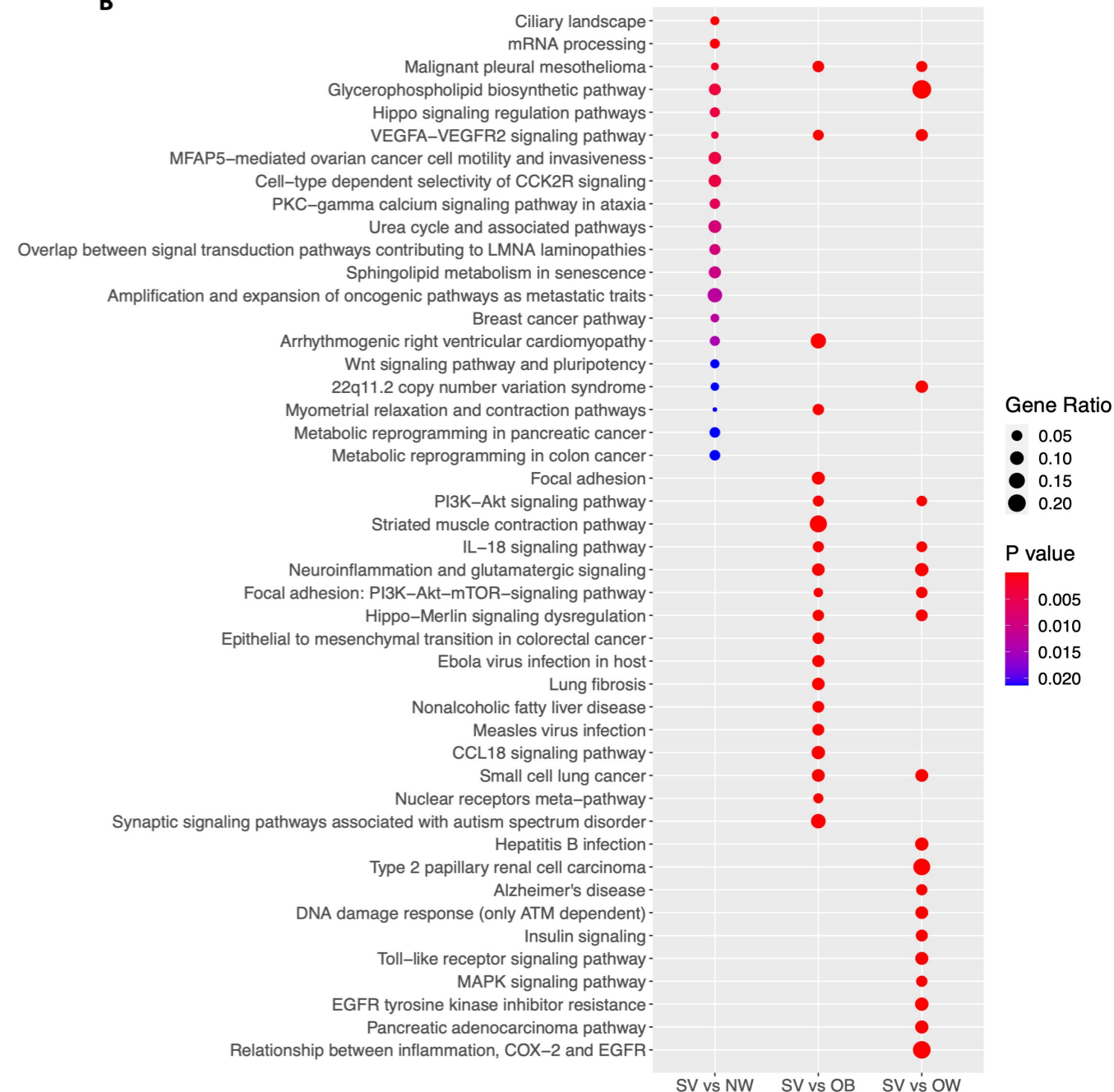

Supplement: Supplementary file 1 [file cells-12-01105-s001.zip › FigureS2_Reactome and WP enrichment analysis.pdf]

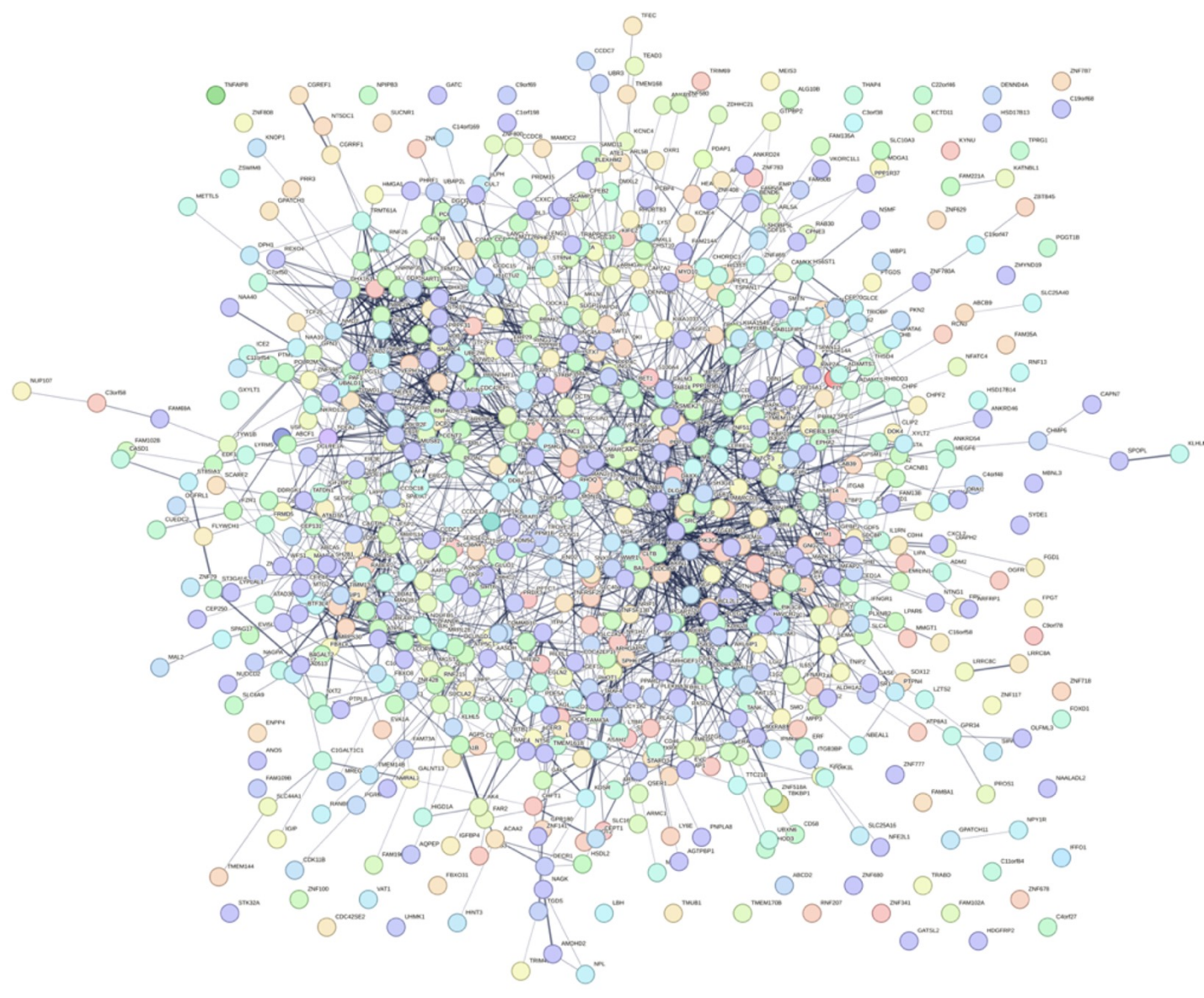

Supplement: Supplementary file 1 [file cells-12-01105-s001.zip › FigureS3_PPI analysis network.pdf]
